# Membrane tubule formation by banana-shaped proteins with or without transient network structure

**DOI:** 10.1038/srep20935

**Published:** 2016-02-11

**Authors:** Hiroshi Noguchi

**Affiliations:** 1 Institute for Solid State Physics, University of Tokyo, Kashiwa, Chiba 277-8581, Japan

## Abstract

In living cells, membrane morphology is regulated by various proteins. Many membrane reshaping proteins contain a Bin/Amphiphysin/Rvs (BAR) domain, which consists of a banana-shaped rod. The BAR domain bends the biomembrane along the rod axis and the features of this anisotropic bending have recently been studied. Here, we report on the role of the BAR protein rods in inducing membrane tubulation, using large-scale coarse-grained simulations. We reveal that a small spontaneous side curvature perpendicular to the rod can drastically alter the tubulation dynamics at high protein density, whereas no significant difference is obtained at low density. A percolated network is intermediately formed depending on the side curvature. This network suppresses tubule protrusion, leading to the slow formation of fewer tubules. Thus, the side curvature, which is generated by protein–protein and membrane–protein interactions, plays a significant role in tubulation dynamics. We also find that positive surface tensions and the vesicle membrane curvature can stabilize this network structure by suppressing the tubulation.

The Bin/Amphiphysin/Rvs (BAR) superfamily proteins regulate the membrane shape of cell organella as well as membrane fusion and fission; therefore, BAR protein dysfunction is implicated in neurodegenerative, cardiovascular, and neoplastic diseases^1^,^2^,^3^,^4^,^5^,^6^,^7^. However, the manner in which these proteins assemble on the biomembrane and cooperate to reshape the membranes is not well understood. The extension of membrane tubes from liposomes and specific adsorption of the BAR superfamily proteins onto tube regions have been observed in *in vitro* experiments^1^,^2^,^3^,^8^,^9^,^10^,^11^,^12^,^13^,^14^,^15^. Frost *et al*. have experimentally determined that F-BAR proteins are adsorbed on flat regions of lipid membranes using electron microscopy^9^. Although the assembly seems to constitute the nucleus of the tubule formation, the tubule protrusion process has not been experimentally observed. Recently, Tanaka-Takiguchi *et al*. reported that the formation dynamics of tubules from a liposome can differ significantly for different F-BAR proteins^13^. That is, FBP17 and CIP4 simultaneously generate many tubule protrusions over the entire liposome surface, while PSTPIP1 and Pacsin2 generate only a few protrusions from a narrow region of the surface. In particular, the tubules induced by CIP4 and PSTPIP1 have the same radius. Thus, the tubule nucleation process depends on the protein type. However, it is not known what causes this difference in tubule nucleation behaviour. Tanaka-Takiguchi *et al*. also reported that the full length of Pacsin2 induces tubulation, but its F-BAR domain region alone does not^13^. In contrast, Wang *et al*. reported the tubulation is induced by F-BAR domain of Pacsin1 more than by the full-length protein^10^.

In the last decade, interactions between laterally isotropic objects on biomembrane, such as transmembrane proteins and adsorbed spherical colloids, have been intensively investigated^16^,^17^,^18^,^19^,^20^,^21^,^22^. In contrast to such studies, however, the interactions between anisotropic adhesives have not yet been explored so far. The BAR domains are banana shaped and generate an anisotropic curvature different from the isotropic spontaneous curvature *C*_0_^23^. This anisotropic nature has recently been receiving increasing theoretical interest. The classical Canham–Helfrich curvature free energy^24^,^25^ has been extended to anisotropic curvatures^26^,^27^,^28^. Dommersnes and Fournier have derived a many-body potential of long-range interactions between point-like anisotropic inclusions and found linear assemblies and egg-carton membrane structures using Monte Carlo simulations^29^,^30^. In addition, the adsorption and assembly of BAR domains have been investigated using atomic and coarse-grained molecular simulations^31^,^32^,^33^,^34^. For example, Simunovic *et al*. have simulated a linear aggregation of N-BAR domains parallel to the domain axis^33^,^34^. However, the relationship between this aggregation and tubulation remains unclear. Further, tubular formation has been simulated using a dynamically triangulated membrane model^35^,^36^ and, also, meshless membrane models^37^,^38^. Despite these numerous advancements, the present understanding of the physics of membrane shape deformation due to anisotropic curvature is still far from complete.

In this paper, we focus on the effects of the spontaneous (side) curvature *C*_side_ of a protein rod perpendicular to its longest axis on the assembly behaviour. The side curvature has not been focused upon in previous studies, but here we reveal that it strikingly changes the assembly dynamics. The excluded volume or van der Waals attraction between proteins and the membrane can effectively generate positive or negative *C*_side_. We simulate flat membranes and vesicles using an implicit-solvent meshless membrane model^38^,^39^,^40^,^41^,^42^, which allows a large-scale simulation. A BAR domain is modelled as a banana-shaped rod, which is assumed to be strongly adsorbed onto the membrane. The rod length corresponds to 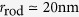 (the BAR domain lengths range from 13 to 27 nm^2^). To investigate the membrane-curvature-mediated interactions, no direct attractive interaction is considered between the rods. Our previous studies showed that parallel and perpendicular assemblies occur separately through membrane-mediated attractive interactions at low protein density^38^, and that polyhedral shapes are formed at high protein density^42^ for vesicles and membrane tubes.

## Results

### Tubulation from Flat Membrane

**Figure 1:**
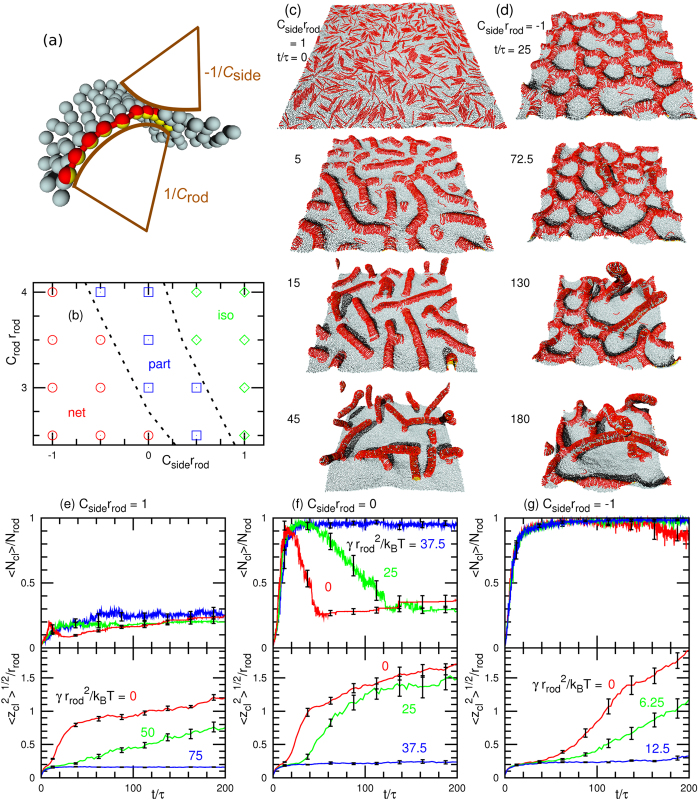
Tubulation dynamics from flat membrane for high rod density, *ϕ*_rod_ = 0.4 (*N*_rod_ = 1,024). (**a**) Protein rod with spontaneous rod and side curvatures, *C*_rod_ and *C*_side_, respectively. The protein rod is displayed as a chain of spheres, the halves of which are coloured red and yellow. The orientation vector lies along the line of the yellow to red hemispheres. The light blue spheres represent membrane particles. (**b**) Dynamic phase diagram of tubulation from a tensionless flat membrane. The red circles represent percolated network formation before tubulation. The green diamonds indicate that the tubules are formed from isolated clusters. The blue squares represent partial network formation, and the dashed lines are guides for the eye. (**c**,**d**) Sequential snapshots of tubulation from tensionless flat membrane for *C*_side_*r*_rod_ = (**c**) 1 and (**d**) −1 at *C*_rod_*r*_rod_ = 4. (**e**–**g**) Time evolution of mean cluster size 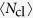 and mean cluster height 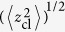 for *C*_side_*r*_rod_ = (**e**) 1, (**f**) 0, and (**g**) −1 at *C*_rod_*r*_rod_ = 4. Error bars calculated from eight independent runs are displayed at several data points.

First, we investigate the tubulation from a tensionless flat membrane (surface tension *γ* = 0) at a high rod density, *ϕ*_rod_ = 0.4 (see Fig. 1). The protein rods are initially equilibrated with the rod curvature *C*_rod_ = 0 and *C*_side_ = 0. Once the spontaneous curvatures are altered at *t* = 0, the rods begin to assemble perpendicularly to the rod axis. For a positive spontaneous curvature of *C*_side_*r*_rod_ = 1 (*C*_side_*C*_rod_ > 0), many tubules simultaneously protrude via the bending of straight rod assemblies (see Fig. 1(c) and Supplemental Movie 1). Branches of the rod network are formed on the membrane for a short time only. When the tubulation is initiated, neighbouring branches are broken through lateral shrinkage of the rod assembly.

For a negative curvature *C*_side_*r*_rod_ = −1, the rods form a percolated network covering the entire membrane area [see the top snapshot in Fig. 1(d)] and a tubule protrudes under membrane undulation (see the second snapshot in Fig. 1(d) and Supplemental Movie 2). Subsequently, tubule growth occurs along the network. Thus, the tubulation dynamics is altered remarkably by a relatively small *C*_side_. Negative and positive *C*_side_ values stabilize and destabilize the network branches, respectively. The tubulation at *C*_side_*r*_rod_ = −1 is significantly slower than that at *C*_side_*r*_rod_ = 1 and much fewer tubules protrude: the average protrusion time of the first tubule are 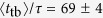 and 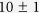 for *C*_side_*r*_rod_ = −1 and 1 at *C*_rod_*r*_rod_ = 4, respectively.

**Figure 2:**
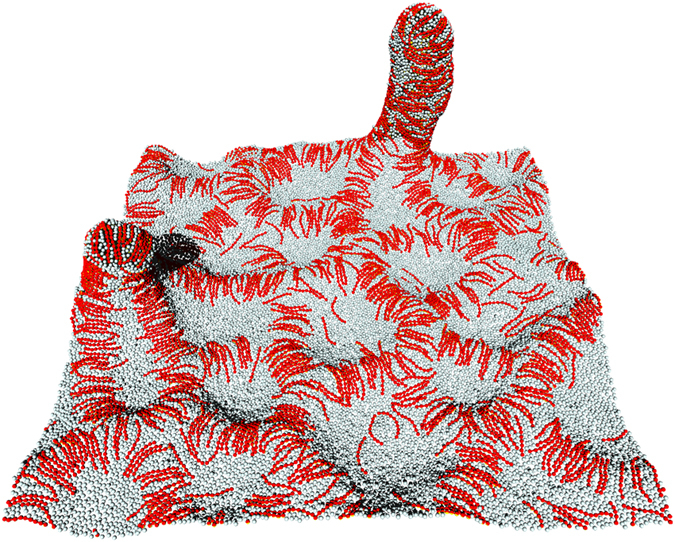
Snapshot of tubulation from tensionless flat membrane at *C*_rod_*r*_rod_ = 2.5, *C*_side_*r*_rod_ = −1, and *ϕ*_rod_ = 0.4.

Such characteristic dynamics is distinguishable from the time evolution of the mean cluster size 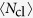 and the root mean square cluster height 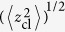, as shown in Fig. 1(e–g). For *C*_side_*r*_rod_ = −1, the majority of the rods belong to one large percolated cluster during the tubulation. In contrast, for *C*_side_*r*_rod_ = 1, 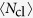 decreases as the tubules are formed and the rod assemblies are divided; subsequently, 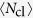 slowly increases owing to tubule fusion. Branched tubules are formed by this fusion [see the bottom snapshot in Fig. 1(c) and the late stage of Supplemental Movie 1]. Based on the evolution of 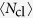, the tubulation pathways are categorized into three groups [see Fig. 1(b)]: tubulation via percolated-network formation (net), via partial-percolated-network formation (part), and without percolation (iso). When a percolated network does not cover the entire membrane surface or a large cluster of 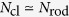 is maintained for a period shorter than 20*τ*, we categorize the tubulation pathway as part. (A typical dynamics is shown in Supplemental Movie 3). For the entire parameter range explored in Fig. 1(b), the final structures are tubules. As *C*_rod_ decreases, the tubulation decelerates and a smaller number of large tubules are formed. The tubules are nucleated and grow from the network vertices at *C*_rod_*r*_rod_ = 2.5 or 0.3 and *C*_side_*r*_rod_ = −1 (see Fig. 2). The tubule radius *R*_tb_ is roughly determined by *C*_rod_ as *R*_tb_ ~ 1/*C*_rod_. At *C*_rod_*r*_rod_ = 4, the tubule with circumference 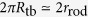 consists of two hemicylinders of the rod assembly.

**Figure 3:**
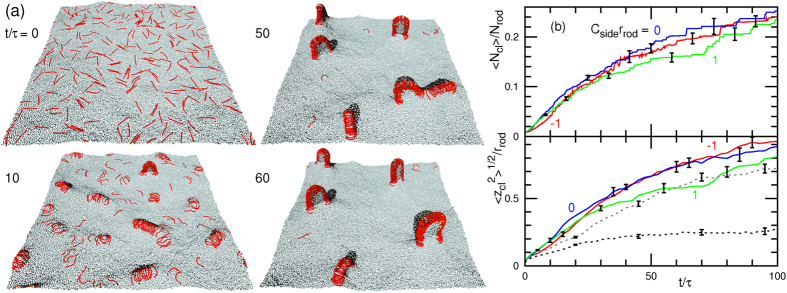
Membrane tubulation from flat membrane for low *ϕ*_rod_ = 0.1 (*N*_rod_ = 256), at *C*_rod_*r*_rod_ = 4. (**a**) Sequential snapshots at *C*_side_ = 0 and *γ* = 0. (**b**) Time evolution of 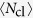 and 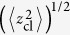. The solid lines represent the data for *C*_side_*r*_rod_ = −1, 0, and 1 at *γ* = 0. The gray and black dashed lines represent the data for 
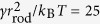
 and 37.5 at *C*_side_ = 0, respectively. Error bars calculated from eight independent runs are displayed at several data points.

Our simulation results show that the network formation suppresses the tubulation. To confirm this more clearly, the effects of *C*_side_ on the rod–membrane interaction are investigated. A percolated network is not formed during the tubulation at a low *ϕ*_rod_ of 0.1. Rather, the rods assemble into linear clusters and, subsequently, the large clusters 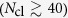 transform into tubules (see Fig. 3 and Supplemental Movie 4). Although the initial cluster formation is slightly slower for negative curvature, i.e., *C*_side_*r*_rod_ = −1, no qualitative difference is detected in the tubulation dynamics [see Fig. 3(b)]. Thus, we conclude that the suppression of the tubulation at high rod density is caused by the mesoscale network formation.

Recent experiments have demonstrated that positive surface tension can suppress tubulation by BAR proteins^15^ and budding by clathrin coats^43^. In our simulation, the positive tension and network formation cooperate to suppress tubulation [see Figs 1(e–g) and 3(b)]. At *ϕ*_rod_ = 0.4, the critical tension decreases with increasing *C*_side_: 
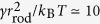
 and 70 for *C*_side_*r*_rod_ = −1 and 1, respectively, where *k*_B_*T* denotes the thermal energy. These are experimentally measurable magnitudes (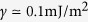 and 0.7 mJ/m^2^, respectively). The assembly of rods into a clustered network is not suppressed by the applied tension. In contrast, network structure breaking does not occur at higher tensions [see Fig. 1(f)]. Thus, the network formation is stabilized by the positive tensions.

When a positive tension *γ* is imposed for the coexisting states of network and tubules as shown in the second snapshot of Fig. 1(d), the tubules continue to grow at 
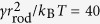
. However, the tubules shrink at 
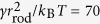
. Thus, at the critical tension 
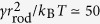
, the tubule elongation force *f*_tb_ by the rod assembly is balanced with the expansion of the projected membrane area by the surface tension as 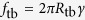. This tension is higher than that required to suppress the tubule protrusion from the flat membranes 
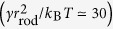
, because a nucleation barrier exists for the protrusion.

### Geometrical Analysis

**Figure 4:**
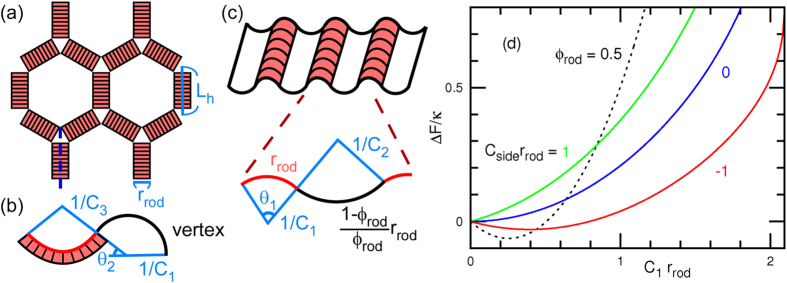
Energy analysis of network structure using simple geometric model. (**a**–**c**) Schematic representation of geometric model. (**a**) Top view of hexagonal array at *C*_1_ = 0. (**b**) Side view of hexagonal array along the dashed line in (**a**). (**c**) Bird’s-eye and front views of striped array. (**d**) Free-energy difference 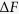 between hexagonal and striped arrays of rod assembly. The solid lines represent the data for *C*_side_*r*_rod_ = −1, 0, and 1 at *ϕ*_rod_ = 0.4. The dashed line represents the data for *C*_side_*r*_rod_ = −1 and *ϕ*_rod_ = 0.5.

To clarify the effects of *C*_side_ on the network formation, the difference between the free energy of a hexagonal array of the rod assembly and that of a striped array is estimated using a simple geometric model (see Fig. 4). Here, a percolated network is modelled as a hexagonal array with side length *L*_h_ [Fig. 4(a)], while an unbranched rod assembly is modelled as a striped array [Fig. 4(c)]. The rod assemblies have rectangular shapes with widths equal to the rod length *r*_rod_. Our analysis shows that the hexagonal network can have lower energy for *C*_side_ < 0. This explains why the membranes are trapped in the branched network as a local free-energy minimum.

In the striped array, the rod assemblies are aligned in parallel with intervals of (1 − *ϕ*_rod_)*r*_rod_/*ϕ*_rod_. The rod assemblies are curved upwards and the other regions are curved downwards, as shown in Fig. 4(c). To maintain the continuity of the normal vector of the membrane, the curvatures have the relation *C*_2_ = *ϕ*_rod_*C*_1_/(1 − *ϕ*_rod_). The curvature energy *F*_st_ of the striped array per the area 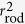 is given by


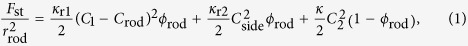


where *κ*_r1_ and *κ*_r2_ are the bending rigidities of the rod assembly parallel and perpendicular to the rod axis, respectively. In our simulation, *κ* = 15*k*_B_*T, κ*_r1_ = 40*k*_B_*T*, and *κ*_r2_ = *κ*^42^.

In the hexagonal array case, the membrane surface is divided into the following three regions: Region I: A rectangular rod assembly with length 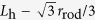 and width *r*_rod_; Region II: A triangular membrane with side length *r*_rod_ at the vertex; and Region III: A hexagonal membrane with side length 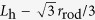. On a flat membrane, the areas of these regions are given by 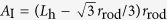, 
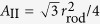
, and 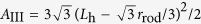, respectively. To simplify the calculation, it is assumed that each region has constant curvatures and its area is independent of these curvatures. Region I has curvatures *C*_1_ along the rod axis and –*C*_3_ perpendicular to the rod axis [along the dashed line in Fig. 4(a)], such that it has a saddle shape. Regions II and III are triangular and hexagonal spherical caps with radii 1/*C*_1_ and 1/*C*_4_, respectively. To maintain the continuity of the normal vector of the membrane, 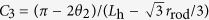 and 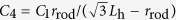. The angle *θ*_2_ is given by 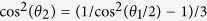, where 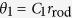, since the vertices of three rod assemblies make contact with each other on the spherical cap and maintain three-fold rotational symmetry. At *θ*_2_ = 0, *C*_1_ has a maximum value of 2*π*/3*r*_rod_. As the area fraction of region I is 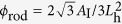, *L*_h_ is obtained as


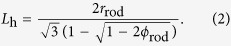


The curvature energy *F*_hex_ of the hexagonal array per 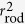 is given by


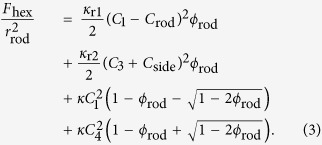


The energy difference 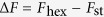 is shown in Fig. 4(d). The first terms in equations (1) and (3) cancel since both arrays have the same curvature *C*_1_ along the rods. Hence, 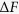 is independent of *C*_rod_ and *κ*_r1_. In the hexagonal array, the curvature energy of the rod assembly is reduced for *C*_side_ < 0, and *F*_hex_ is smaller than *F*_st_ for small *C*_1_. Thus, the branched network can be stabilized by a negative *C*_side_ during the formation of the rod assembly.

In our simulation, networks are formed in a wider range of *C*_side_ for smaller values of *C*_rod_ [see Fig. 1(b)]. This dependence can be explained by an effective increase in the area fraction *ϕ*_rod_. The rod assembly region contains more membrane particles with decreasing *C*_rod_ (see light blue particles in rod network and tubules in Fig. 2). Thus, the area of region I increases. At *ϕ*_rod_ = 0.5, the minimum of 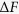 is twice that at *ϕ*_rod_ = 0.4, although the area fraction is only 25% larger [see the dashed line in Fig. 4(d)]. Thus, the effective increase in the rod region enhances the network formation.

As *ϕ*_rod_ decreases, *L*_h_ of the hexagonal array increases [see equation (2)]. However, it is difficult for the hexagonal network with long *L*_h_ to form spontaneously, since the tubule formation begins before the rod assembly reaches *L*_h_. The simulation results for *ϕ*_rod_ = 0.1 indicate tubulation from clusters with 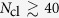 at 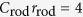. Thus, the formation threshold of the percolated network is 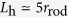, where 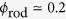. In the simulation at *ϕ*_rod_ = 0.2, the clusters are typically percolated only in one direction at *C*_rod_*r*_rod_ = 4 and *C*_side_*r*_rod_ = −1, which supports this estimation of the critical density.

### Shape Transformation of Small Vesicles

**Figure 5:**
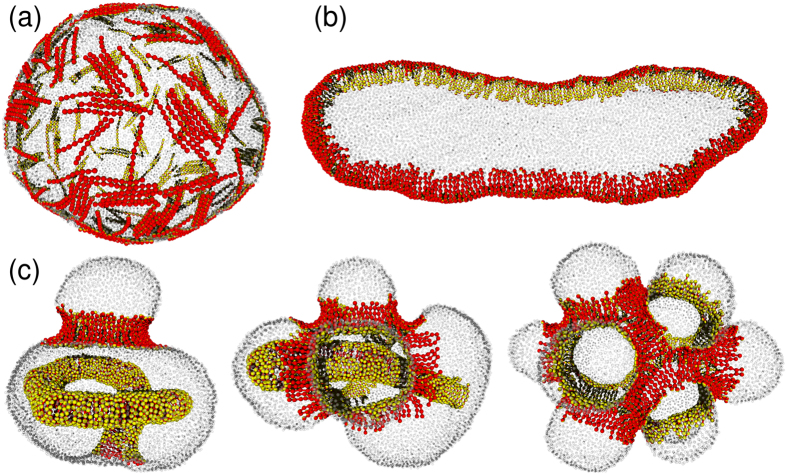
Snapshots of vesicles at *ϕ*_rod_ = 0.3 and *N*_rod_ = 288. (**a**) Spherical shape in thermal equilibrium at *C*_rod_ = 0 and *C*_side_ = 0. (**b**) Elongated discoidal shape in thermal equilibrium at *C*_rod_*r*_rod_ = 4 and *C*_side_*r*_rod_ = −1. (**c**) Three metastable shapes at *C*_rod_*r*_rod_ = −4 and *C*_side_*r*_rod_ = 1. The membrane particles are displayed as small transparent spheres for clarity.

Next, we investigate the tubulation from a vesicle of radius *R*_ves_ = 3.07 *r*_rod_ at *ϕ*_rod_ = 0.3 (see Fig. 5) and demonstrate that the original membrane curvature *C*_ves_ = 1/*R*_ves_ = 0.33/*r*_rod_ changes the tubulation dynamics. For the positive curvature *C*_rod_*r*_rod_ = 4, no tubulation is obtained for *C*_side_*r*_rod_ = −1, 0, and 1. Instead, the vesicle deforms to an elliptic disk and the rods surround the disk rim [see Fig. 5(b)]. A discoidal bud is often transiently formed, but the rearrangement of the rod assemblies results in disk formation even for *C*_side_*r*_rod_ = −1 (see Supplemental Movie 5). Thus, outward tubulation and network formation are suppressed in small vesicles.

In contrast, for the negative curvature *C*_rod_*r*_rod_ = −4, tubulation into the inside of the vesicle is obtained (see Fig. 5(c) and Supplemental Movie 6). A percolated network with *C*_side_*C*_rod_ < 0 has a significantly longer lifetime than in the flat membrane case [see the middle and right snapshots in Fig. 5(c)]. The coexistence of tubules and a ring is also obtained [see the left snapshot in Fig. 5(c)]. The ring stabilizes an outward bud. For *C*_rod_ < 0, the rods bend the membrane towards the interior (opposite to the original membrane curve), such that the rods locally form a saddle shape in which the two principal curvatures have opposite signs. The network and ring structures are stabilized by the positive (opposite) *C*_side_ > 0, but not by *C*_side_ < 0.

## Discussion

We have revealed that, in addition to the spontaneous curvature along the protein rods *C*_rod_, the perpendicular spontaneous curvature *C*_side_ significantly influences the protrusion of membrane tubules. The percolated-network structure of the rod assembly has a long lifetime for *C*_side_ < 0, because the saddle membrane shape at branches of the rod network is stabilized by the opposite curvature of *C*_side_ with respect to *C*_rod_. Thus, the network formation decelerates the tubulation significantly, despite having a minor effect on the equilibrium property. Both positive surface tensions and membranes originally bending in the same direction as *C*_side_ < 0 can stabilize the network structure. Our findings provide new insights into the regulation of biomembrane shapes by curvature-inducing proteins.

Here, we employ Langevin dynamics, in which hydrodynamic interactions are neglected. Since the static stability of the network branch is the key factor, we do not expect the obtained *C*_side_ dependence to be qualitatively changed by the hydrodynamic interactions. However, the network formation condition may be modified. The diffusion coefficient of the proteins on the membrane depends on the protein size, and fast protein diffusion compared to the membrane deformation speed likely enhances the network formation.

The F-BAR domain of Pascin is considered to have a nonzero side curvature, since it has an S-shape on the membrane surface in the addition to the curvature perpendicular to the membrane^10^. Pascin induces membrane tubes in a wide range of diameter^10^,^14^. In the present simulations, we did not obtain such a behaviour. It may require a larger side curvature or attractive interactions between the rods. The rod assembly with large side curvatures is an interesting problem for further studies.

An assembly of F-BAR proteins, Cdc15, has been observed along the contractile ring of cell division^44^. Their adsorption to the inner leaflet of the plasma membrane is considered to yield a ring structure similar to that shown in Fig. 5(c). Our study suggests that the side curvature may play an important role in the formation of neck-like structures during cell division and membrane budding in endo/exocytosis.

## Methods

We employ one of the meshless membrane models^41^, in which a fluid membrane is represented by a self-assembled one-layer sheet of membrane particles. A membrane particle has an excluded volume with diameter *σ* and an orientational degree of freedom. The solvent is implicitly accounted for by an effective potential between the membrane particles. The mechanical properties of the fluid membrane can be varied over a wide range. The details of the meshless membrane model and protein rods are described in refs 38,41, respectively. In this study, we employ the parameter set used in ref. 38 for a membrane with isotropic spontaneous curvature *C*_0_ = 0. The membrane has mechanical properties typical for lipid membranes: Bending rigidity *κ*/*k*_B_*T* = 15 ± 1, tensionless membrane area per particle 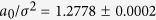, area compression modulus 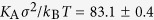, and edge line tension 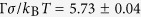.

A BAR protein is modelled as a curved rod consisting of a chain of *N*_sg_ membrane particles with *r*_rod_ = 10*σ* and *N*_sg_ = 10. The rod has anisotropic spontaneous curvature *C*_rod_ along its length and spontaneous curvature *C*_side_ perpendicular to its length. When two protein rods come into contact, *C*_side_ is applied between the rods. When the rod is surrounded by membrane particles, the spontaneous curvature *C*_side_/2 is applied between the rod and neighbouring membrane particles. A molecular dynamics with a Langevin thermostat is employed^41^,^45^. The simulation results are displayed with a time unit of 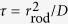, where *D* is the diffusion coefficient of the membrane particles in the tensionless membranes. We use total particle numbers *N* = 25,600 and 9,600 for flat membranes and vesicles, respectively. The rod density is defined as *ϕ*_rod_ = *N*_rod_*N*_sg_/*N*.

The mean square cluster height 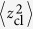 is calculated as follows. A rod is considered to belong to a cluster when the distance between the centres of mass of the rod and one of the rods in the cluster is less than *r*_rod_/2. The height variance of each cluster is calculated as 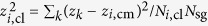, where *N*_*i*,cl_ is the number of rods belonging to the *i*-th cluster, *z*_*i*,cm_ is the *z* component of the centre of mass of the cluster, and the summation is taken over all rod segments in the cluster. Finally, 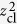 is calculated as the average of 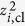 for all clusters.

## Additional Information

**How to cite this article**: Noguchi, H. Membrane tubule formation by banana-shaped proteins with or without transient network structure. *Sci. Rep.* **6**, 20935; doi: 10.1038/srep20935 (2016).

## Supplementary Material

Supplementary Information

Supplementary Information

Supplementary Information

Supplementary Information

Supplementary Information

Supplementary Information

Supplementary Information
