# Supplementary material for: Membrane tubule formation by banana-shaped proteins with or without transient network structure
Source: Sci Rep. 2016 Feb 11;6:20935. doi: 10.1038/srep20935 (PMC4750063; doi:10.1038/srep20935)
Supplement: Supplementary Information [file srep20935-s1.pdf]

Supplemental Material: Membrane tubule formation by banana-shaped proteins with or without transient network structure

Hiroshi Noguchi
Institute for Solid State Physics, University of Tokyo, Kashiwa, Chiba 277-8581, Japan. e-mail: noguchi@issp.u-tokyo.ac.jp

Movie Captions

Supplemental Movie 1: Tubulation from a tensionless membrane at high rod density $\phi_{\rm rod} = 0.4$, $C_{\rm rod}r_{\rm rod} = 4$, and $C_{\rm side}r_{\rm rod} = 1$.

Supplemental Movie 2: Tubulation from a tensionless membrane at $\phi_{\rm rod} = 0.4$, $C_{\rm rod}r_{\rm rod} = 4$, and $C_{\rm side}r_{\rm rod} = -1$.

Supplemental Movie 3: Tubulation from a tensionless membrane at $\phi_{\rm rod} = 0.4$, $C_{\rm rod}r_{\rm rod} = 4$, and $C_{\rm side} = 0$.

Supplemental Movie 4: Tubulation from a tensionless membrane at low rod density $\phi_{\rm rod} = 0.1$, $C_{\rm rod}r_{\rm rod} = 4$, and $C_{\rm side} = 0$.

Supplemental Movie 5: Formation of a discoidal vesicle at $\phi_{\rm rod} = 0.3$, $C_{\rm rod}r_{\rm rod} = 4$, and $C_{\rm side}r_{\rm rod} = -1$.

Supplemental Movie 6: Tubule formation to the inside of a vesicle at $\phi_{\rm rod} = 0.3$, $C_{\rm rod}r_{\rm rod} = 04$, and $C_{\rm side}r_{\rm rod} = 1$.
